# Supplementary material for: Evidence supported by Mendelian randomization: impact on inflammatory factors in knee osteoarthritis
Source: Front Med (Lausanne). 2024 May 28;11:1382836. doi: 10.3389/fmed.2024.1382836 (PMC11165061; doi:10.3389/fmed.2024.1382836)
Supplement: Supplementary file 1 [file Data_Sheet_1.ZIP › data/GCST90274780_5e6/780.leaveoneout.pdf]

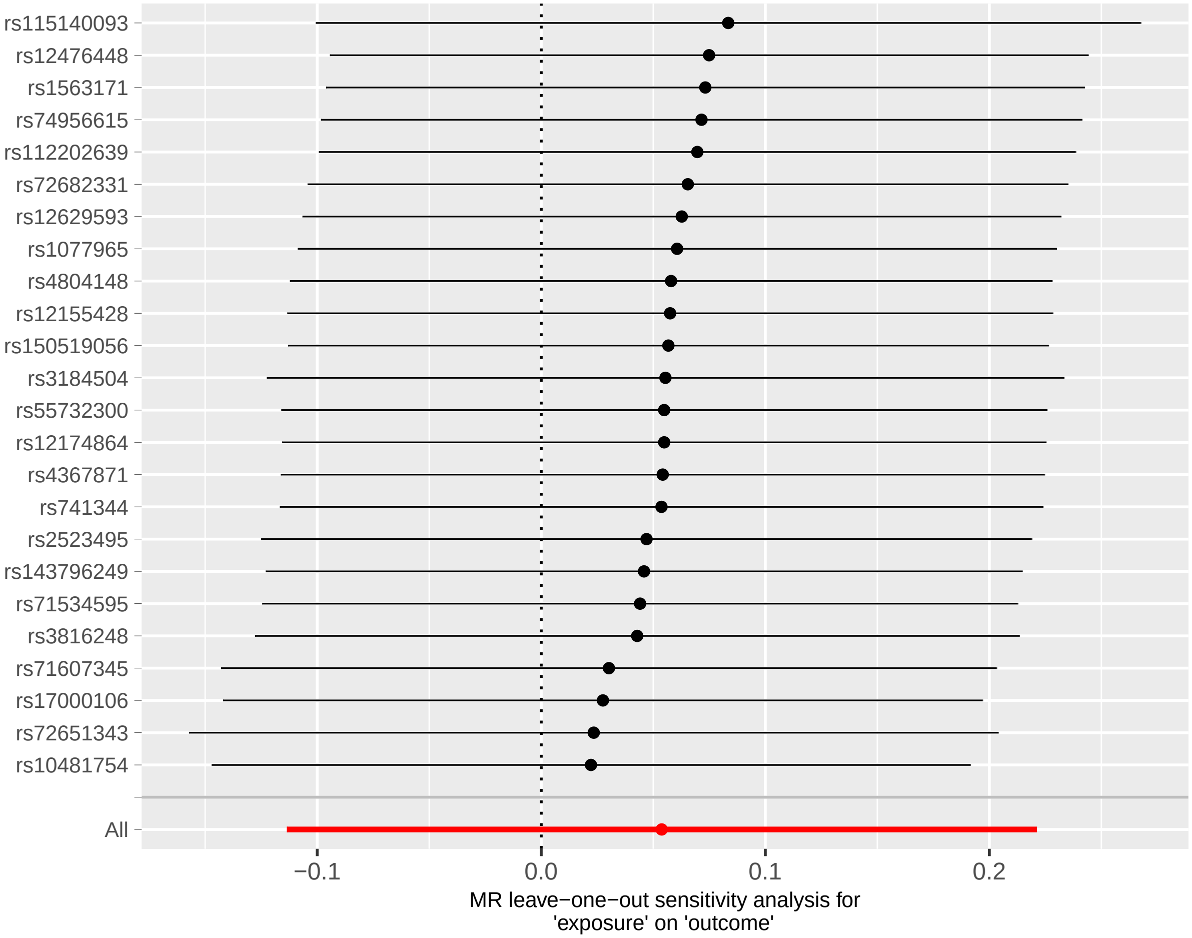

rs115140093
rs12476448
rs1563171
rs74956615
rs112202639
rs72682331
rs12629593
rs1077965
rs4804148
rs12155428
rs150519056
rs3184504
rs55732300
rs12174864
rs4367871
rs741344
rs2523495
rs143796249
rs71534595
rs3816248
rs71607345
rs17000106
rs72651343
rs10481754
All
−0.1
0.0
0.1
0.2
MR leave−one−out sensitivity analysis for
'exposure' on 'outcome'
